# The Louisville Medical Club

**Published:** 1855-06

**Authors:** John Bartlett

**Affiliations:** Sec’y.


### THE LOUISVILLE MEDICAL CLUB.

The Louisville Medical Club met at the house of Dr. Knight, on the evening of the 1st of May, 1855. Dr. L. Rogers, Senior Vice-President in the chair, and the following members present:—Drs. Knight, Wible, Marshall, Hardin, L. P. and D. W. Yandell, Palmer, Gross and Bartlett.

In compliance with the request of the Club, Dr. Benjamin R. Palmer expressed his views on peritoneal inflammation.

He said that his attention had been particularly called to this disease. It prevailed where he had practiced in Vermont. He had been educated in the antiphlogistic school, and accordingly his first patient was bled three times—she died. The next patient he bled three times in twenty-four hours without affording relief. At this juncture he chanced upon a copy of John Bell's journal, containing lectures from Armstrong, who stated that for peritoneal inflammation there were three remedies—blood-letting, opium and calomel. He gave the opium in five grain, and the calomel in one grain doses. Of the two remedies, he deemed opium the more efficacious. Dr. Palmer's second case began to improve when opium was given.

About ten years ago having been called to a patient, rather advanced in the disease, he *commenced* the treatment with opium and calomel. He then experimented to determine if blood-letting might not be dispensed with—and he succeeded without it.

Dr. Clark tried opium alone, at Bellevue, and found it competent to cure. This was about ten years ago, and since that time Dr. Palmer has treated peritoneal inflammation with opium. He is satisfied of its extraordinary curative effects. He gives it to the extent of rendering the patient comfortable—two grains every two hours in ordinary cases. Three years ago he treated a case giving five grains of opium every two hours for three days. In two or three instances he narcotised his patients, so that in one case the respirations were only three and a half in a minute. If the pulse of patients without opium is 120, it will sometimes fall to 100 under its influence. This remedy has been successful in his hands when there was hiccup, vomitings, pinched face, &c.

Cases of peritoneal inflammation are rare even in the neighborhood of Woodstock; but for the past twelve years he has treated three or four cases each year. Some of the physicians in his section purged their patients—frequently calling the disease bilious colic. Such treatment was unsuccessful.

In relation to the constipating effect of opium, he had observed that as soon as the violence of the disease abates, there are frequent actions on the bowels. He remembers a stout man who went twenty-one days, another nineteen, and others twelve and fourteen days without evacuations.

Dr. Clark has satisfied himself that opium was competent to the cure of this inflammation by employing it during a severe epidemic of puerperal fever at Bellevue. Dr. Clark has given enormous doses of opium. In one case *four hundred and seventy-two* grains were given; in another one ounce was exhibited in fourteen days.

Dr. Palmer mentioned the case of a woman who recovered from a terrific attack, and in whom he is confident the peritoneal adhesions gave way after having troubled the patient for several years. She referred the breaking up of these adhesions to the jarring produced by equestration; it was sudden and attended with pain, shock, &c.

Sometimes the opium produces perspirations; when it does he simply diminishes—does not suspend the dose. He has never known effusion in the cranium to result from its use.

In cases of colic Dr. Palmer gives opium first, and then uses evacuants.

Dr. Clark should be considered in the first rank of antiphlogistic practitioners, and he proposed to try opium in pericarditis.

Dr. Palmer had never encountered an epidemic with opium. Dr. Clark, however, had; of seventeen of his cases, eleven recovered; those that did not recover were among the first treated. As to its modus operandi he had not speculated; its interference with the due oxygenation of the blood might be considered a mode.

Dr. John Hardin reported three cases treated by opium at the suggestion of Dr. Palmer.

One was a woman with metroperitonitis—sick two days. She had been treated with purgatives. Her pulse was 130; her abdomen enormously distended, and her face hippocratic. He ordered her one grain of opium every hour. She slept, and the next morning he commenced giving one half grain of morphine every two hours, continuing this dose for several days. She recovered in eight days.

Last winter he had another case complicated with severe bronchitis. She had bad symptoms, tympanitis—in short, looked as if she would die in twelve hours. Under the opium treatment she recovered in ten days.

In a third case occurring in a stout woman, he gave nothing throughout but opium; she was well in ten days.

Dr. Hardin has seen a number of cases of peritonitis occur during epidemics of erysipelas. He remembers that of two cases one died. The treatment adopted was not mentioned.

Dr. Gross referred to the mechanical effects of opium, as explanatory of its curative powers in peritonitis.

Dr. Hardin enumerated its physiological effects.

Dr. Knight said that he treated a woman with peritonitis twenty years ago with opium, nitre and ipecac. He was in the habit of giving it as a remedy in these cases

Dr. L. P. Yandell extolled its curative powers. He considers it the best remedy for sick headache.

Dr. David W. Yandell reported that the scurvy was pretty abundant in the Louisville Marine Hospital, as well as in the poorer quarters of the city. The worst cases were those who had labored for a year or more on the railroads, deprived of vegetable food. He remarked that they had no special predilection for a vegetable diet. He was treating them with acidulated drinks, and a full allowance of vegetables.

Dr. Wible proposed some questions as to the connexion between rheumatism and dyspepsia. Dr. D. W. Yandell remarked that the connexion was recognized and fully set forth by Dr. Fuller in his work on Rheumatism.

Dr. Lewis Rogers was requested to give a history of the scurvy as now prevailing in Kentucky, at the next meeting.

(After accepting the invitation of Dr. Rogers to meet at his house on Tuesday evening, the 5th of June next, the Club adjourned.) JOHN BARTLETT, Sec'y.

